# Ischemic Heart Disease and Heart Failure: Role of Coronary Ion Channels

**DOI:** 10.3390/ijms21093167

**Published:** 2020-04-30

**Authors:** Paolo Severino, Andrea D’Amato, Mariateresa Pucci, Fabio Infusino, Lucia Ilaria Birtolo, Marco Valerio Mariani, Carlo Lavalle, Viviana Maestrini, Massimo Mancone, Francesco Fedele

**Affiliations:** Department of Clinical, Internal, Anesthesiology and Cardiovascular Sciences, Sapienza University of Rome, Viale del Policlinico, 155-00161 Rome, Italy; paolo.severino@uniroma1.it (P.S.); damatoandrea92@gmail.com (A.D.); puccimariateresa@gmail.com (M.P.); fabio.infu@gmail.com (F.I.); ilariabirtolo@gmail.com (L.I.B.); marcoval.mariani@gmail.com (M.V.M.); carlo.lavalle@uniroma1.it (C.L.); viviana.maestrini@uniroma1.it (V.M.); massimo.mancone@uniroma1.it (M.M.)

**Keywords:** heart failure, ischemic heart disease, microcirculation, ion channel, coronary artery disease, coronary microvascular dysfunction

## Abstract

Heart failure is a complex syndrome responsible for high rates of death and hospitalization. Ischemic heart disease is one of the most frequent causes of heart failure and it is normally attributed to coronary artery disease, defined by the presence of one or more obstructive plaques, which determine a reduced coronary blood flow, causing myocardial ischemia and consequent heart failure. However, coronary obstruction is only an element of a complex pathophysiological process that leads to myocardial ischemia. In the literature, attention paid to the role of microcirculation, in the pathophysiology of ischemic heart disease and heart failure, is growing. Coronary microvascular dysfunction determines an inability of coronary circulation to satisfy myocardial metabolic demands, due to the imbalance of coronary blood flow regulatory mechanisms, including ion channels, leading to the development of hypoxia, fibrosis and tissue death, which may determine a loss of myocardial function, even beyond the presence of atherosclerotic epicardial plaques. For this reason, ion channels may represent the link among coronary microvascular dysfunction, ischemic heart disease and consequent heart failure.

## 1. Introduction

Heart failure (HF) is a complex syndrome responsible for high rates of death and hospitalization among the general population worldwide. One of the most frequent causes of HF is ischemic heart disease (IHD), which leads to the loss of myocardial tissue and contractile force [1]. Coronary artery disease (CAD) determines a myocardial reduction in oxygen supply, which causes an impairment of myocardial contraction and relaxation [2,3]. In this context, the “modern” cardiology focuses much of its attention on the study of epicardial atheromatous plaque, its etiology, its prevention and its diagnostic and therapeutic interpretation. As early as the 1970s, the effects of progressive narrowing, due to a stenosis, on coronary flow, at rest and at maximum levels, have been described [4]. In fact, a reduction in the diameter of a coronary artery ≥50% limits its maximum vasodilatory capacity, while a reduction ≥85% determines a reduction in flow, even at rest. The pathophysiological continuum between obstructive atherosclerosis of the epicardial coronary arteries, myocardial ischemia and HF is now well defined. Angiographic data have confirmed the relationship between the severity and the extent of coronary atherosclerotic disease and survival [5]. However, in the literature, several studies suggest that coronary obstruction is only an element of a complex multifactorial pathophysiological process that leads to myocardial ischemia [6]. Furthermore, it is also known that abnormalities, in the function and structure of the coronary microcirculation, are linked to various clinical conditions [2,3]. An insufficient interest in coronary microcirculation and its pathophysiological role is devoted in clinical practice. What does this vascular network, made up of coronary arterioles with a diameter between 50 and 200 microns, really represent? Does a cardiologist really have to take this into account in complex decision-making? 

## 2. Coronary Blood Flow and its Regulation Mechanisms: Role of Ion Channels

From a physiological point of view, the oxygen consumption of myocardial tissue is about 75–80% at rest [7]. Consequently, in conditions of increased metabolic demand by the myocardium, the main compensation mechanism is represented by the Coronary Flow Reserve (CFR) [2,3,4,7,8,9]. This represents the ability of the coronary circulation to dilate in response to the increased requests, expressed as the ratio between the maximum amount of flow in hyperemia, with respect to its value in resting conditions [2,3,4,7,8,9]. In reality, in conditions of extreme physical exercise, the increase in flow may not fully compensate for the increase required, thus, a further increase in oxygen extraction is necessary [7]. In this context, it is useful to remember that in the coronary microcirculation about 60% of the coronary resistance is determined [2,3,4,7,8,9]. There are several regulatory mechanisms for CFR: (i) the endothelial, (ii) the nervous, (iii) the myogenic and (iv) the metabolic mechanisms [2,3,4,7,8,9]. (i) The coronary endothelium acts through a variety of vasoactive substances, known as endothelium-derived hyperpolarizing factor (EDHF) [7,10,11]. EDHF represents a class of vasodilators that produce hyperpolarization, and thus, release smooth muscle cells, by inhibiting voltage-gated Ca^++^ channels. In coronary microcirculation, the endothelium determines flow-dependent vasodilation, which is mainly mediated by nitric oxide (NO) [7,10,11]. This is formed by the endothelial NO Synthase Enzyme (eNOS), in response to acetylcholine and *shear stress*, or tangential stress, causing dilation of vascular smooth muscle cells (VSMCs) [7,10,11]. These actions are mediated by the increase in cyclic guanosine monophosphate (cGMP) and the consequent activation of potassium Ca^++^-activated channel (KCa) and adenosine triphosphate-sensitive potassium (K_ATP_) channels [2,3,4,7,8,9,10,11]. In addition, asymmetric dimethylarginine (ADMA), an inhibitor of the endogenous NOS enzyme, is a known risk factor for cardiovascular diseases, through the impairment of the synthesis of NO by eNOS [12,13]. (ii) The nervous mechanisms of regulation of resistance to the coronary vascular network depend on the orthosympathetic and parasympathetic nervous system [7,14,15,16]. The first works as a vasoconstrictor through the α-receptors present on the epicardial vessels and as a vasodilator through the β-receptors of the intramyocardial vessels and the KCa channels [7,14,15,16]. Instead, the parasympathetic system acts through acetylcholine, which causes vasodilation, through the production of NO by eNOS, in physiological endothelial conditions [7,10,11,12,13,14,15,16,17]. (iii) Myogenic mechanisms act through coronary self-regulation systems that keep blood flow constant, despite changes in perfusion pressure in response to the increasing tone of the coronary VSMCs. The increase in myogenic tone depends on the Ca^++^-dependent signal through the L-type Ca^++^ channels and the voltage-gated potassium (Kv) channels [7,18]. (iv) Changes in coronary flow have a greater effect on the larger caliber arterioles and on the small arteries, while the release of vasoactive metabolites mainly affects the small arterioles and capillaries [3,4,7,8,19,20]. The metabolic regulation works through adenosine, adenosine triphosphate (ATP), adenosine diphosphate (ADP), prostaglandins and reactive oxygen species (ROS), by dilating arterioles with a diameter of less than 100 microns [3,4,7,8,19,20]. An imbalance between supply and demand of oxygen leads to adenosine-induced vasodilation, mainly by stimulating K_ATP_ channels [7,19,20]. In fact, in normal conditions, creatine kinase inhibits adenylate kinase and, through inhibition by ATP, the K_ATP_ channels are mostly in a “closed” state [7,19,20]. Hypoxia, on the other hand, reduces the activity of creatine kinase and increases the adenylate kinase activity [7,19,20]. This causes the production of adenosine monophosphate (AMP), the opening of the K_ATP_ channels, hyperpolarization of the membrane and coronary vasodilation [3,4,7,8,19,20]. 

Coronary blood flow (CBF) regulation, at the microcirculatory level, is mainly controlled by the myocardial metabolic demand [3,4,7,8,9,18,19,20]. Therefore, an alteration of the regulation mechanisms leads to a dysfunction of coronary vasomotor tone and myocardial ischemia, beyond the presence of atherosclerotic plaque in epicardial vessels [3,4,7,8,9,21,22,23].

However, there is no clear consensus about the real role of the systems responsible for the cross talk between coronary flow and myocardial metabolism [3,4,7,8,9,21,22,23].

In literature, the central role of coronary ion channels in metabolic coronary vasodilation has been described [3,4,7,8,9,18,24,25,26]. In fact, as described, in acute stress conditions, such as myocardial ischemia, the physiological CBF compensatory response and CFR are linked to numerous coronary ion channels activity [3,4,7,8,9,18,24,25,26]. The vascular smooth muscle tone is, in fact, regulated by the membrane potential, which controls the amount of Ca^++^ in the sarcoplasm through the voltage-gated Ca^++^ channels [3,4,7,8,9,18,24,25,26]. Membrane hyperpolarization, through the opening of K^+^ channels in smooth coronary muscle cells, reduces the activation of L-type Ca^++^ channels, leading to a reduction in intracellular Ca^++^ and vasodilation [7,14,15,16,24,25,26]. On the other hand, the closure of the K^+^ channels leads to the depolarization of the membrane and causes vasoconstriction [7,14,15,16,24,25,26]. Three major classes of K^+^ channels have been identified in coronary vasculature (endothelial and VSMCs): K_ATP_ channels, KCa channels and Kv channels [3,4,7,8,9,14,15,16,18,24,25,26]. Numerous other ion channels have been described in the complex architecture of coronary vasomotor tone, such as Ca^++^, sodium (Na^+^) and Transient Reception Potential (TRP) ion channels [3,4,7,8,9,14,15,16,18,24,25,26]. 

## 3. Coronary Microcirculation in the Pathophysiology of Ischemic Heart Disease and Heart Failure

IHD, and in particular CAD, represents the main cause of HF [27,28]. However, CAD, and in particular the presence of an atherosclerotic plaque in epicardial coronary arteries, does not always determine myocardial ischemia and, on the other hand, myocardial ischemia is not always justified by the presence of an atherosclerotic plaque. In CAD patients, myocardial systolic dysfunction has been described as the main pathophysiological mechanism involved in HF [27,28]. Classically, patients with IHD who develop HF have a clinical history of myocardial infarction with atherosclerotic disease of epicardial arteries, as shown by coronary angiography [27,28]. However, the absence of atherosclerotic plaques assessed by coronary angiography cannot exclude the presence of coronary microvascular dysfunction (CMD) as pathophysiological mechanism of HF [27,28]. In these patients, a prevalent diastolic dysfunction has been proposed [27]. Moreover, CMD may represent the pathophysiological substrate of left ventricular diastolic dysfunction [27,28].

In the literature, an increasing number of studies underline the central role of CMD in the pathophysiology of IHD and HF, beyond atherosclerotic disease [3,4,8,9,29,30]. Moreover, CMD represents one of several pathophysiological mechanisms, which may cause type II myocardial infarction [31]. CMD is due to an impairment of microvascular endothelial and non-endothelial adaptation of CBF to myocardial metabolic demands and it may be associated with myocardial ischemia, independently from CAD [3,4,7,8,9,10,21,22,23]. Impairment of mediators of CBF regulation, such as coronary ion channels, may lead to CMD. Moreover, CMD, which alters hemorheological features of CBF, may promote atherosclerotic plaques development in epicardial vessels, through the increase of shear stress and the prolonged exposition of coronary vessels wall to low density lipoproteins (LDL), ROS, inflammation mediators and advanced glycation end-products (AGEs) [3,4,7,8,9,10,21,22,23]. There are several methods through which CMD can be assessed. Transthoracic echocardiogram, cardiac magnetic resonance and positron emission tomography (PET) scan can be used to evaluate CFR non-invasively, while coronary angiography can be used to assess it invasively [28,31]. During coronary angiography, intracoronary administration of acetylcholine and adenosine may be used to evaluate endothelial dependent and independent vasodilation respectively. CMD is defined by a CFR < 2. 0 [28,31]. 

Over the past few years, some authors have hypothesized the central role for CMD in the pathophysiology of HF and in myocardial remodeling [29,30,31,32]. 

Moreover, other authors have suggested a link between CMD, through endothelial dysfunction, and severity of symptoms in HF patients [32,33]. According to ALLAHAT trial and MEDIA (The Metabolic Road to Diastolic Heart Failure) European registry for HF, a high body mass index is often seen in these patients and cardiovascular risk factors, such as arterial hypertension, diabetes mellitus and dyslipidemia may lead to HF through microvascular dysfunction. Endothelial dysfunction is associated with a reduced bioavailability of NO and reduced activity of K_ATP_. Moreover, CMD stimulates cardiomyocytes hypertrophy, fibrosis and microvascular rarefaction, which are the main histological alterations seen in HF [32,34,35,36].

Additionally, Paulus et al. [34] and Franssen et al. [37] focused on the possible role of CMD in the pathophysiology of HF. They identified a possible sequence of events that may bring HF. All the cardiovascular risk factors contribute to a systemic pro- inflammatory state. In HF patients, there is a high tumor necrosis factor alfa (TNF-α), interleukin 6 (IL-6), pentraxin 3 and ST2 blood concentration [34,38,39]. However, systemic inflammation is not predictive for the risk to develop HF [34,40]. At microcirculation, the inflammatory state, but also each risk factor directly, may cause a higher production of ROS, an increased expression of chemokines and selectins, such as vascular cell adhesion protein 1 (VCAM-1) and E-selectin, mitochondrial function impairment and reduced NO availability [37]. Moreover, a physical training program seems to determine an upregulation of eNOS, beyond its impact on ion channels, improving symptoms and exercise tolerance in HF patients [34,41]. NO deficiency reduces protein kinase G activity, which, together with lower Phospholamban phosphorylation, induced by ROS, causes the persistence of high levels of Ca^++^ in cardiomyocytes, endothelial cells and smooth muscle cells, increasing the cardiac wall stiffness and coronary vasoconstriction [34,42]. Inflammation leads to fibrosis and cardiomyocytes hypertrophy [34,43]. Myocardial hydrogen peroxide (H_2_O_2_) and superoxide anions are significantly increased in HF. There are several sources of ROS such as mitochondria, eNOS, xanthine oxidase and nicotinamide adenine dinucleotide phosphate hydrogen (NADPH) oxidase, NOX 2 and 4. In HF patients, NOX2 is upregulated in coronary endothelial cells and not in cardiomyocytes, focusing on the role of microvascular dysfunction as *primum movens* in the pathological alteration and cardiac remodeling in HF [37,44]. 

In this context, some authors distinguish patients with preserved and reduced left ventricular ejection fraction (LVEF), as suggested by the European Society of Cardiology (ESC) classification of HF [27]. However, in the study of pathophysiology of HF, several limitations of using the mere LVEF are described in literature [45,46,47,48,49]. In fact, LVEF is not clearly associated with clinical features and pathophysiological mechanisms related to HF [45,46,47,48,49]. LVEF is not enough to distinguish diastolic from systolic dysfunction [45,46,47,48,49]. Moreover, LVEF does not consider the complexity of HF, which is a multiorgan syndrome [45,46,47,48,49]. For this reason, other classification to stage HF, more completely, are proposed. Regarding this, HLM classification, which follows the TNM classification used to stage cancers, seems to be interesting [45,46,47,48]. The parameter “H” defines the heart damage, “L” the involvement of lungs and “M” the malfunction of peripheral organs. HLM classification provides an evaluation of all organs involved in HF, integrating instrumental, clinical and laboratory parameters [45,46,47,48]. 

## 4. Ion Channels in Ischemic Heart Disease and Heart Failure

Ion channels are end-effectors of CBF regulation mechanisms and they have a central role in the adaptation of CBF in response to cardiomyocytes metabolism, through the continuous modulation of coronary vascular tone. They modulate the state of contraction and relaxation of VSMCs and the endothelial function [4,7]. For this reason, the impairment of their function, which may be genetically determined or acquired, as a consequence of the exposition to cardiovascular risk factors, represents an important mechanism, which may lead to CMD. As previously described, CMD may represent a cause of myocardial infarction [28,31]. It may lead to diastolic dysfunction in HF [27]. Moreover, an involvement of ion channels and CMD in the ischemia and diastolic dysfunction related to several cardiomyopathies, such as hypertrophic and restrictive cardiomyopathies, have been described [23,30,32,33,34,35,36,37]. Ion channels are also expressed by cardiomyocytes where they are involved in the regulation of myocardial contractility and excitability [7,50]. 

Several studies demonstrate the central role of dysfunctional ion channels in the determinism of IHD through CMD, also independently from CAD, and in HF pathophysiology [3,4,8,9,10,21,22,23,29,32,33]. Moreover, several genetic variants about coronary and cardiac ion channels encoding genes are also associated to IHD and HF, beyond cardiovascular risk factors [9,51]. For this reason, ion channels have been studied as a target for IHD, and for reduced contractility and arrhythmias in HF [52]. Overall, abnormal levels of intracellular Na^+^ [52,53,54], downregulation of K^+^ [52,55,56] channel and Ca^++^ cycling defects have the most important effects in HF determinism.

### 4.1. ATP-Sensitive Potassium (K_ATP_) Channel 

K_ATP_ channels are expressed by cardiomyocytes, coronary endothelial and smooth muscle cells, where they regulate myocardial contractility and relaxation and coronary vascular tone, through the regulation of intracellular Ca^++^ concentration [57].

K_ATP_ channels belong to the Kir channel family (inward rectifiers). K_ATP_ are molecular biosensors capable of translating changes in intracellular metabolism into responses in membrane excitability, in order to maintain homeostasis.

Through close integration with cellular metabolic pathways, coronary K_ATP_ channels have a well-defined role in maintaining cardiac performance, under stress conditions. The cardioprotective properties of K_ATP_ channels are underlined by studies with knock-out animal models for the genes coding for the regulatory subunits (Kir 6.x and SURx) of the channel, which make the heart more vulnerable to ischemic damage leading to HF. Furthermore, the inhibition of the function of K_ATP_ channels leads to a greater susceptibility to pathological Ca^++^-dependent remodeling, the progression of organ failure and death [58,59,60], with the result that K_ATP_ channels are necessary for the adaptive response of the heart during stress. Studies in men reveal additional cardiac vulnerability to stress factors, mediated by genetic variations of K_ATP_ channels. In fact, several studies have correlated genetic polymorphisms, capable of determining a dysfunction in the ionic channel, linked with HF, susceptibility to dilated cardiomyopathy and electrical instability [61,62,63]. In particular, a higher frequency of a particular polymorphism for the K_ATP_ channel has been described in patients with HF [61] able to alter its function [62,63]. In fact, the correct functioning of the K_ATP_ channels is essential in humans for optimal cardiac performance, during an increasing metabolic demand, caused by physical exercise, and in case of myocardial energy deficit, induced by HF.

Several genetic variants of K_ATP_ may associate CMD with IHD and HF. Knockout mice for a SUR2 subunit showed higher risk to develop coronary vasospasm and arterial hypertension, while a missense mutation, A1513T, and frameshift mutation (Fs.1524), expressed in heterozygosity, about SUR2A, were associated with reduced myocardial systolic function in dilated cardiomyopathy [64]. In patients with IHD and chronic HF, a steady and aerobic physical training is important for rehabilitation. It improves cardiac function and the quality of life, reducing mortality [57]. Kraljevic et al. [57] demonstrated that physical exercise increases the expression of the K_ATP_ subunit SUR2A, protecting cardiomyocytes against ROS damage and improving their contractile function. Moreover, the inhibition of K_ATP_, through the use of Glibenclamide, is associated with a reduction of intracellular Ca^++^ handling induced by exercise, demonstrating the close association between intracellular Ca^++^ concentration, improving cardiac contractile function and K_ATP_ activity [57].

Physical exercise is involved in the protection of myocardium from ischemia and in the recovery of fail cardiomyocytes, after myocardial ischemia, because it determines an increase in myocardium contractility, through a reduction of action potential duration of cardiomyocytes. According to the increase of myocardial metabolism and storage of AMP in cardiomyocytes, K_ATP_ function increases progressively [65,66,67]. Wang et al. demonstrated an upregulation of cardiomyocytes K_ATP_ expression and function and a reduced action potential duration, induced by regular physical exercise [65]. This mechanism is related to a quick repolarization, reduced intracellular Ca^++^ overload and a prolonged diastolic time with reduced energy consumption for cardiomyocytes, mainly at high heart rate [65]. 

In several tissues, among which myocardium, ischemia, determined by the interruption of blood flow, and the quick reperfusion define ischemia-reperfusion injury, in which ROS play a central role. During myocardial infarction, ischemia reperfusion injury is the main mechanism that leads to necrosis and apoptosis [68]. Ischemic preconditioning is an important myocardial mechanism against ischemia reperfusion injury [68]. Before a prolonged state of ischemia, there are small periods of transient ischemia and reperfusion, which trigger several protective mechanisms, leading to ischemic preconditioning [68]. Among them, a transient accumulation of a sub-lethal levels of ROS has been described [68]. Moreover, Hypoxia-inducible factor 1 (HIF-1) may play an important role in the regulation of myocardial protective genes expression, in the late phase of ischemic preconditioning [68]. However, ROS have a central role in cardiomyocytes death in several pathological conditions, such as IHD and HF.

The mitochondrial permeability transition (MPT) induction represents a central mechanism in cardiomyocytes’ death [69]. Mitochondrial permeability transition represents a big channel permeable to several ions and molecules and it is associated with mitochondrial membrane potential dispersion [69]. It may be involved in mitochondrial Ca^++^ homeostasis, in physiological condition [69]. MPT may be activated by ROS production, causing the dispersion of mitochondrial membrane potential and triggering a burst generation of ROS [69]. This important mechanism, which may lead to cell death, explaining the coupling between ROS and MPT, is defined by ROS-induced ROS release [69]. Moreover, it couples long chains of mitochondria in the same cell and its development and reversibility depends on the antioxidants cell systems function [69]. In cardiomyocytes, NO may stabilize thiol groups, in conditions of strong oxidative stress, playing a role against MPT induction and apoptosis [69]. 

Several studies have shown the central role of eNOS and NO up-regulation in cardioprotective effects of late preconditioning [70,71,72]. 

NO has a central role in the adaptive response of coronary vascular tone to myocardial metabolism and in myocardial contractile response [70,71,72]. During cardiac ischemia, NO production is increased because of the activation of Inducible Nitric Oxygen Synthase (iNOS). There is a link between mitochondrial K_ATP_ and iNOS activity in mice [70]. Mitochondrial K_ATP_ is activated by NO production induced by iNOS, during ischemia. Moreover, NO induces up-regulation of iNOS activity, through nuclear factor κ light chain enhancer of activated B cells (NFκB) [70]. 

In case of IHD, during reperfusion, the release of hydroxyl radical (OH), from mitochondria, [73,74,75] determines a contractile dysfunction known as myocardial stunning [72,75]. Moreover, OH plasmatic levels, in IHD patients, are linked to the risk to develop HF, in patients with acute myocardial ischemia [73,76]. In order to prevent reperfusion damages and myocardial stunning, activation of mitochondrial K_ATP_ channels determines stabilization of mitochondrial membrane and reduction of OH release and, therefore, failing myocardial protection against OH- induced damage [73,77,78]. However, mitochondrial K_ATP_ channels opening may lead to increasing ROS production and release because they provoke mitochondrial matrix alkalization, involving complex I and III [79]. Moreover, blocking K_ATP_ channels with sulfonylurea worsens left ventricular function [73]. In HF patients, the genetic variants E23K and I337V of *KCNJ11*, encoding for Kir6.2 subunit, has been related to end-diastolic volume and mass of the left ventricle [80]. Moreover, regarding the same gene *KCNJ11*, the single nucleotide polymorphism rs5215_G/G seems to represent a protective factor against IHD, independently from cardiovascular risk factors [9,81].

Several studies have shown the role of K_ATP_ as target in the treatment of IHD and acute and chronic HF [82,83,84]. Several molecules, such as Levosimendan and Nicorandil, induce coronary vasodilation through the opening of K_ATP_, improving CBF, myocardial metabolism and contractility [82,83,84]. They also have a beneficial effect on other organs whose functions are compromised in HF as kidney, lungs and liver [82,83,84]. 

### 4.2. Potassium Calcium-Activated Channel (KCa) Channels 

KCa channel family consists of small (SKCa), intermediate (IKCa) and large conductance (BKCa) channels [7]. KCa channels regulate the efflux level of K^+^ to maintain ideal levels of membrane potential in VSMCs and endothelial cells, resulting in an appropriate vascular tone [7]. The impaired function of all three subtypes of KCa may be associated with a dysregulation of coronary vascular tone, in response to myocardial oxygen and metabolites supply [7]. 

In physiological conditions, the hyperpolarization of coronary endothelial cells and following vasodilation are determined by an increase of intracellular Ca^++^ and vasoactive substances release, mediated by IKCa and SKCa channels [7]. The same two channels’ subtypes, not BKCa channels, are involved in thrombin-related endothelium dependent vasodilation [7]. In addition, bradykinin induces coronary endothelium dependent vasodilation, through SKCa and BKCa channels [7]. H_2_O_2_-induced coronary vasodilation is mediated by BKCa, IKCa and SKCa channels and, in diabetic patients, the endothelial dysfunction seems to be associated with the impairment of SKCa and IKCa channels [7]. In patients affected by heart disease, coronary dilation, mediated by adenosine, involves SKCa channels [7]. 

BKCa channels are also involved in the neural mechanism of CBF regulation. These subtypes of channels are activated in relation to β-adrenoceptors stimulation by catecholamines [7].

Oliván-Viguera et al. [85] studied the coupling between IKCa3.1 activity and endothelial dysfunction, in cardiovascular disease and diabetes. Using SKA-121 and SKA-111, which are strong positive modulators of the IKCa3.1 channel, an improved cells’ hyperpolarization and endothelial dependent coronary vasodilation, induced by bradykinin, has been showed in rats [85]. It underlines the potential role of IKCa3.1 as therapeutic target against endothelial dysfunction [85]. 

Mishra et al. [86] tested the role of SKA-31, a positive modulator of SKCa2.3 and IKCa3.1, on coronary endothelial dependent vasodilation, in diabetes mellitus. SKA 31 restored the vasodilatory response to bradykinin and adenosine. Therefore, through SKA-31, SKCa2.3 and IKCa3.1 reduce coronary vascular tone, increasing CBF also in advanced phases of diabetes mellitus [86]. These channels determine VSMCs relaxation because they have a role in endothelial hormone-related production of NO, together with an hyperpolarizing effect, which reaches VSMCs through myo-endothelial gap junctions [86]. 

BKCa channels are involved in the response of smooth coronary muscle cells to endothelial stimulation, providing an important negative feedback mechanism to vasoconstrictor responses [87]. Moreover, BKCa channels are activated by the increase in intracellular Ca^++^ concentration and membrane depolarization. These channels have a decisive role in adjusting the depolarization response and in balancing coronary vasoconstriction. In fact, multiple vasoconstrictive substances inhibit BKCa channels, such as angiotensin II, endothelin and thromboxane A2. BKCa channels have been identified as effectors of vasodilatation induced by the phospholipase A2 and lipoxygenase metabolites [88]. Other studies have highlighted the role of BKCa channels in H_2_O_2_-induced coronary vasodilation [89,90,91]. Furthermore, in metabolic syndrome, CMD is related to the decrease in the function of BKCa channels, in VSMCs [89]. The reduced function of the BKCa channel leads to inappropriate coronary vasoconstriction [89,90,91] and, consequently, to dysfunction of the microcirculation. BKCa channel is also involved, in the animal model, in cardioprotection, through preconditioning, after short ischemic damages. In fact, blocking the BKCa channel deletes the cardioprotection mechanisms [90]. In addition, it has been shown that Peroxynitrite (ONOO-), consisting of the interaction of superoxide with NO, inhibits physiological vasodilation, mediated by BKCa channels, expressed on VSMCs of human coronary arterioles [91].

In arterioles of CAD patients, Nishijima et al. demonstrated a shift from a Kv1.5-BKCa to a predominant BKCa vasodilation, induced by H_2_O_2_ [92]. BKCa expression is reduced by high glucose levels and it is increased in several pathological conditions, such as atherosclerosis and hypertension, in order to compensate for reduced Kv1.5 expression [92].

Plasma aldosterone levels are associated with cardiovascular mortality, also independently from cardiovascular risk factors. Moreover, a gain of function of renin-angiotensin-aldosterone system (RAAS) has been associated with several cardiovascular pathological conditions, such as hypertension, myocardial infarction and HF. Aldosterone mediates vascular and cardiac remodeling associated with these diseases. Recently, Khan et al. focused on the role of aldosterone in the determinism of microvascular dysfunction and related condition, such as IHD and HF. Independently from arterial blood pressure and metabolic alterations, aldosterone reduced coronary adenosine induced vasodilation, determining downregulation of several K^+^ channels, among which KCa. It may reduce the expression of genes *KCNN3* and *KCNN4* encoding for SKCa2.3 and IKCa1, respectively. For this reason, aldosterone antagonists may have a specific role against CMD associated with IHD and HF [93].

Cyclic adenosine monophosphate (cAMP) is one of the main molecules in determining vasodilation and guaranteeing cardiac perfusion. The synthesis of cAMP is linked to the activity of the Adenylyl Cyclase, while its degradation takes place through the activity of the phosphodiesterases (PDEs), in particular PDE3 and PDE4 [94,95,96,97]. BKCa channels activation determines VSMCs relaxation and they are activated by increasing intracellular Ca^++^ levels, linked to Ca^++^ influx or the activation of ryanodine receptors, and by cAMP signaling [95,98,99]. BKCa channel activity is reduced in many animal models of HF [94,98]. The relationship among cAMP-PDE-BKCa has a central role in vascular tone regulation and inhibition of PDE3 and PDE4 improved vasodilation, in coronary circulation [95]. Sildenafil is a PDE5 inhibitor, but it also has many effects on different mechanism linked to cardio-protection [100]. Sildenafil works by activating mitochondrial ATP-sensitive K^+^ channels, determining mitochondrial membrane stabilization especially during ischemic-reperfusion injury [100]. Mitochondrial KCa, located on the inner membrane of mitochondria, are also activated by sildenafil and they are involved in myocardial protection, by reducing Ca^++^ overload in cardiomyocytes and hyperpolarizing mitochondrial membrane [100,101]. 

### 4.3. Voltage-Gated Potassium (Kv) Channels 

The family of Kv channels have a central role in the regulation of metabolism, through an oxidation-reduction process [3,4,102]. In this way, mitochondrial H_2_O_2_ production, linked to the metabolism through a feed-forward mechanism, controls the vascular tone, by opening redox-sensitive Kv channels [103]. A study has shown that H_2_O_2_-dependent vasodilation is mediated by Kv channels [103]. Different types of Kv channels are expressed in cardiac and coronary tissue, although Kv1.5 seems to play a major role in smooth muscle cells. It has been observed that Kv1.5 mice -/- have insufficient metabolic vasodilation and that the imbalance between metabolism and coronary flow leads to cardiac dysfunction [104]. The Kv1.5 channels are, in fact, critical for metabolic dilation, in the coronary circulation [105]. In addition, during hyperglycaemia states, peroxynitrite production damages the Kv channels, compromising CBF and myocardial perfusion, which can chronically lead to heart muscle failure [106]. Liu et al. demonstrated that a reduced coronary independent vasodilation could be explained also by an impair of Kv channels, mediated by ROS and AGEs, in diabetes. ROS and AGEs induce VSMCs damage, causing inflammation, through NF-κB pathway. Moreover, the reduction of AGEs and the inhibition of NF-κB pathway improve CBF regulation, contrasting CMD [107]. Nishijima et al. focused on the role of Kv1 family channels in vasodilation and its possible different impact on arteriolar blood flow regulation, between CAD and non-CAD patients [92]. In arterioles of CAD patients, they found a reduced H_2_O_2_-related vasodilation. It may be due to a reduced VSMCs surface expression of Kv1.5 channels, induced by ROS, which seems to mainly impair the membrane protein trafficking. Those mechanisms may be involved in the microvascular dysfunction associated with CAD [92]. Ohanyan et al. studied the possible role of the Kv1.3 coronary ion channel, in the metabolic cross talk between CBF and cardiomyocytes’ metabolic demand [108]. These authors observed a reduced vasodilatory response in Kv1.3 knock out mice and in those treated with correolide, a Kv1.3 channel blocker, during increasing myocardial work [108]. Moreover, the Kv1.3 channel is involved in H_2_O_2_ coronary related vasodilation, but not in acetylcholine- and adenosine-induced vasodilation [108]. An impairing of the Kv1.3 channel may be associated with CMD, although there is an absence of specific genetic studies which confirm this relationship in human [108].

Berwick et al. focused on the electromechanical interaction between Kv and the voltage-gated L-type Ca^++^ channel Cav1.2, in coronary VSMCs, and its influence on CBF regulation [109]. Obesity and metabolic syndrome could reduce Kv channel expression and function, increasing Cav1.2 activity, causing intracellular Ca^++^ overloading with significant vasoconstriction [109]. According to Berwick et al., this mechanism may contribute to CMD in obesity [109]. Moreover, the administration of nifedipine, a Ca^++^ channel blocker, reduced microvascular resistances and improved the adaptation of CBF relatively to myocardial oxygen consumption (MVO_2_) in obese, but not in lean swine [109].

Hypertension and diabetes mellitus reduce Kv7 channel expression by arterial VSMCs, a condition that contributes to microvascular dysfunction in these pathological conditions. In a study by Morales-Cano et al., the enhancement of Kv7 channel, through the administration of a peroxisome proliferator-activated receptor-β/δ (PPARβ/δ) agonist, improved coronary microvascular tone, during a hyperglycemic state [110]. A PPARβ/δ agonist could protect the expression of genes *KCNQ 1, 4* and *5*, which encode the Kv7 channel, which was severely compromised by hyperglycemia [110].

Although atrial and ventricular arrhythmias are not always associated with the presence of myocardial dysfunction, ischemic scar or ion channels alterations [111], they have a great impact on mortality in IHD and HF patients [112]. However, several alterations in cardiac ion channels, related to HF, have been described [113,114].

Sridhar et al. studied Kv atrial channels in chronic HF and their role in the HF-related atrial fibrillation [113]. They demonstrated an unchanged atrial myocardium Kv4.3 subunit expression, but increased K^+^ currents mediated by Kv4, due to an overexpression of Kv channel-interacting protein 2 (KChIP2) [113]. KChlP2 overexpression is involved in regional atrial myocardium differences of transient outward K^+^ current (I_to_), in HF [113]. Moreover, it is involved in Kv1.5 membrane trafficking, which may be one of the causes of a reduced Kv1.5 expression they observed [113]. These mechanisms may be involved in the reduction of atrial outward K^+^ current 4-aminopyridine sensitive (I_Kur_), seen in HF [113]. Suzuki et al. showed a downregulation of Kv4.2 in mice with HF induced by dilated cardiomyopathy [114]. Left ventricular Kv4.2 expression, in HF mice, was 60% compared to the wild type model by the first month and it reduced to 25% by the second month [114]. 

### 4.4. Transient Receptor Potential (TRP) Channels 

TRP channels represent a big family of ion channels expressed by several cells, among which endothelial and VSMCs of coronary arteries and cardiomyocytes [115,116]. TRP channels are permeable to several cations and they are mainly involved in the Ca^++^ currents regulation, through the cell membrane [115,116]. They have a role in several cell functions, such as proliferation, migration, differentiation, relaxation and contraction [115,116]. Several stimuli are able to activate TRP channels, such as ROS, shear stress, paracrine factors, phospholipids and mechanical stress [115,116]. Moreover, in the cardiovascular system, their role is even more important in pathophysiological conditions. Several studies underline TRP channels involvement in the pathophysiology of myocardial hypertrophy and remodeling, arrhythmias, arterial and pulmonary hypertension, CMD, atherosclerosis, IHD and HF [115,116]. Several types of TRP channels are expressed in blood vessels and in coronary arteries. TRP channels are involved both in microvascular dysfunction and in atherosclerosis [115,116]. TRPA1, TRPV1-4 and TRPC1-6, are expressed on the endothelial cell surface and they allow the passage of Ca^++^, regulating endothelial dependent vasodilation in response to several molecules, such as EDHF, NO and prostacyclin [115,116]. Kochukov et al. demonstrated a role in endothelial dependent vasodilation for TRPC1 and 3; indeed, knockout mice for these channels showed a reduced aortic vasodilation, in response to carbachol [116,117] According to Willette et al., the administration of a TRPV4 agonist, GSK1016790A, determined vasodilation by EDHF and a consequent reduction of arterial blood pressure [117,118]. Freichel et al. demonstrated that knockout mice for TRPC4 showed a reduced vasodilatory response to acetylcholine [115,119]. Earley et al. showed that the TRPV4 channel was involved in the coupling of endothelial dependent vasodilation and VSMCs relaxation, through the interaction with BKCa and ryanodine receptor [116,120]. TRPM4 and 7, TRPC1, 5 and 6, andTRPV1, 2 and 4 are expressed by VSMCs, and they are involved in the myogenic regulation of vascular tone [116,121]. Knockout mice for TRPM4 showed a greater vascular tone and they were hypertensive [115,122], while through the inhibition of TRPC6, a reduced VSMCs contraction has been demonstrated [123].

TRP channels may also have a role in atherosclerosis and CAD. Wei et al. found that TRPV1 was involved in the progression of atherosclerotic process, in apolipoprotein E knockout mice [116,124]. Allowing a better endothelial dependent vasodilation, through the channel gain of function, the single nucleotide polymorphism I957V of TRPC4 could have a protective role against myocardial ischemia [116,125]. TRPC1, 3, 4 and 6 are involved in post-ischemic angiogenesis in myocardium. This process represents important compensatory mechanisms after myocardial ischemic injury, which directly involves microcirculation and it is mainly sustained by vascular endothelial growth factor (VEGF). TRP channels contributes to Ca^++^ influx, as a consequence of the coupling between VEGF and its receptors, allowing endothelial cells proliferation and migration. TRP channels are also associated with cardiac remodeling, fibrosis and HF, after myocardial infarction [115,116]. TRP channels, modulating intracellular Ca^++^ concentrations, seem to be associated with ischemia reperfusion injury and preconditioning [115,116].

## 5. Conclusions

**Figure 1:**
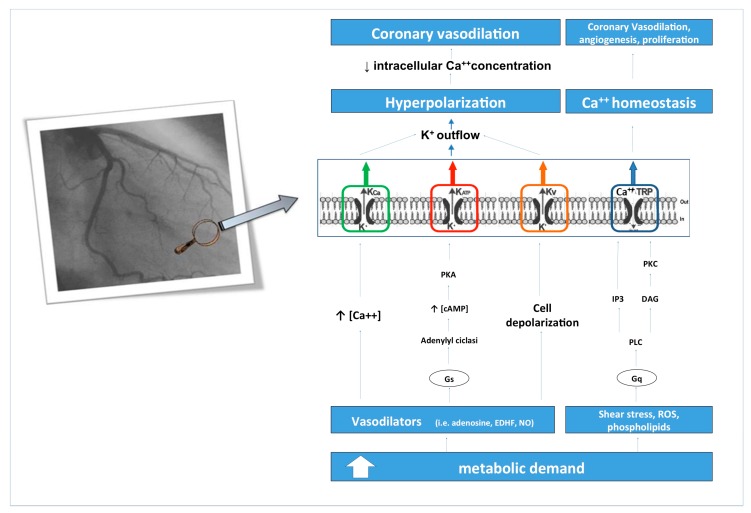
Represents the role of coronary ion channels matching coronary flow to metabolic demands, in coronary microcirculation. An increasing myocardial metabolic request requires an increase in coronary flow, through the action of various regulators in coronary microcirculation. As the metabolic demand increases, the coronary ion channels determine the hyperpolarization of the membrane and the closure of voltage-dependent Ca^++^ channels. As a consequence, the concentration of intracellular Ca^++^ decreases, and this leads to a decreasing tone of the coronary smooth muscle cell, resulting in the vasodilatory response. Abnormality function or expression of these ion channels, disturbing the communication between metabolism and coronary microcirculation, can lead to heart disease, ischemia and heart failure. KCa: potassium Ca^++^-activated channel; K_ATP_: ATP-sensitive potassium channels; Kv: voltage-gated potassium channels; TRP: transient receptor potential; cAMP: cyclic adenosine monophosphate; PKA: protein kinase A; EDHF: endothelial derived hyperpolarizing factor; NO: nitric oxide; ROS: reactive oxygen species; PLC: phospholipase C; IP3: inositol trisphosphate; DAG: diacylglycerol; PKC: protein kinase C.

Myocardial blood flow adaptation to different metabolic conditions is essential for normal myocardial function. This adaptive process requires a complex system of factors. Several mechanisms are involved in myocardial flow regulation, including metabolic and neurohumoral factors and physical influences, such as changes in intraluminal pressure or effects induced by shear stress on the vessel wall. In this context, the role of coronary ion channels is crucial in matching CBF to metabolic demands (Figure 1). Due to their role in repolarization, in coronary vascular cells (endothelial and smooth muscle), changes in the expression or activity of ion channels often result in vascular tone abnormalities. Thus, pathophysiological conditions, characterized by the development of vascular hyperactivity, including arterial hypertension, dyslipidemia, diabetes mellitus and genetic variations, such as mutations or polymorphisms, can lead to alterations in the expression or function of coronary ion channels. Furthermore, anomalies in the activity of these channels caused by ROS, during diabetes-induced oxidative stress, cause dysfunction in vascular resistance control. This damages the regulatory system dependent on myocyte metabolism, which inevitably leads, in the long term, to the development of coronary artery microcirculation dysfunction and myocardial insufficiency. This can be considered a new paradigm in the pathophysiology of HF, whereby an inability of the coronary circulation to satisfy the metabolic demands of the heart, due to microcirculation dysfunction and its regulatory mechanisms, including ion channels, leads to the development of hypoxia, fibrosis and tissue death, which eventually results in a loss of myocardial function, even beyond the atherosclerotic epicardial plaque [3,4,45,46,47,48,126,127,128]. In addition, current therapies used in HF, such as beta-blockers, ACE inhibitors and aldosterone antagonists, reduce myocardium oxygen demand and reduce dysfunction effect in metabolic vasodilation. The imbalance between oxygen supply and demand, due to an alteration of cardiac microcirculation and coronary ion channels, is even more evident with high levels of cardiac work. Thus, pharmacological interventions, able to reduce cardiac work, also minimize the reduction of the effects of microcirculation dysfunction, and they probably slow the progression of the disease. In recent years, scientific literature has turned its attention towards the study of coronary microcirculation and its regulators, including ion channels, also with regard to the pathophysiological continuum that links microcirculatory dysfunction to IHD and HF. However, further research is still needed to shed light on this intriguing, yet still unexplored aspect.
